# Incremental health expenditure and lost days of normal activity for individuals with mental disorders: results from the São Paulo Megacity Study

**DOI:** 10.1186/s12889-015-2099-1

**Published:** 2015-08-05

**Authors:** Alexandre Dias Porto Chiavegatto Filho, Yuan-Pang Wang, Antonio Carlos Coelho Campino, Ana Maria Malik, Maria Carmen Viana, Laura Helena Andrade

**Affiliations:** Department of Epidemiology, School of Public Health, University of São Paulo, São Paulo, Brazil; Institute of Psychiatry, University of São Paulo Medical School, São Paulo, Brazil; Department of Economics, School of Economics, Business Administration and Accounting, University of São Paulo, São Paulo, Brazil; Department of Business Administration, Fundação Getúlio Vargas, São Paulo, Brazil

**Keywords:** Health expenditure, Mental disorders, Depression, Anxiety, Days of normal activity, Metropolitan region, Brazil

## Abstract

**Background:**

With the recent increase in the prevalence of mental disorders in developing countries, there is a growing interest in the study of its consequences. We examined the association of depression, anxiety and any mental disorders with incremental health expenditure, i.e. the linear increase in health expenditure associated with mental disorders, and lost days of normal activity.

**Methods:**

We analyzed the results from a representative sample survey of residents of the Metropolitan Region of São Paulo (*n* = 2,920; São Paulo Megacity Mental Health Survey), part of the World Mental Health (WMH) Survey Initiative, coordinated by the World Health Organization and performed in 28 countries. The instrument used for obtaining the individual results, including the assessment of mental disorders, was the WMH version of the Composite International Diagnostic Interview 3.0 (WMH-CIDI 3.0) that generates psychiatric diagnoses according to the Diagnostic and Statistical Manual of Mental Disorders (DSM-IV) criteria. Statistical analyses were performed by multilevel generalized least squares (GLS) regression models. Sociodemographic determinants such as income, age, education and marital status were included as controls.

**Results:**

Depression, anxiety and any mental disorders were consistently associated with both incremental health expenditure and missing days of normal activity. Depression was associated with an incremental annual expenditure of R$308.28 (95 % CI: R$194.05-R$422.50), or US$252.48 in terms of purchasing power parity (PPP). Anxiety and any mental disorders were associated with a lower, but also statistically significant, incremental annual expenditure (R$177.82, 95 % CI: 79.68–275.97; and R$180.52, 95 % CI: 91.13–269.92, or US$145.64 and US$147.85 in terms of PPP, respectively). Most of the incremental health costs associated with mental disorders came from medications. Depression was independently associated with higher incremental health expenditure than the two most prevalent chronic diseases found by the study (hypertension and diabetes).

**Conclusions:**

The fact that individuals with mental disorders had a consistent higher health expenditure is notable given the fact that Brazil has a universal free-of-charge healthcare and medication system. The results highlight the growing importance of mental disorders as a public health issue for developing countries.

## Background

Mental disorders have been growing in both absolute and relative terms among chronic diseases and are now considered a leading cause of disability in the developing world [1]. Its rapid increase has been attributed to a number of conditions frequently found in poor regions, such as low educational attainment, persisting unemployment, relative material disadvantage, gender and racial discrimination, humanitarian crises, social isolation and violence [2]. Recent reports have called for international action to reduce social and economic disparities which are the cause as well the consequence of mental disorders [3, 4].

An important issue with studying the distribution of mental disorders in developing countries is the absence of appropriate diagnosis and care. A report by the World Health Organization (WHO) found that almost half of the world’s population lives in a country where there is one or less psychiatrist for every 200,000 individuals [4]. While for Brazil that number is 1.5, for countries with high per capita income the median is 17.2. The difficulty in finding appropriate care leads to the inability of detecting the presence of mental disorders and therefore to the underreporting of the real prevalence of mental disorders in developing regions.

There is a growing interest in identifying the individual and environmental factors associated with depression and anxiety, but the socioeconomic consequences of mental disorders are still unclear, especially in developing regions [5]. Recent international studies have shown that mental disorders are associated with both higher direct health expenditures and lost days of normal activity [6, 7]. A recent study from the US found an annual excess health expenditure of $1,657.52 for individuals with anxiety disorders, mostly due to medications and medical visits [8]. Another study using US data found that individuals with serious mental disorders had reduced annual earnings of $16,306, after controlling for individual factors such as age, gender, race and urbanicity [9]. Despite the consistent results found for the US on mental disorders and higher financial costs, it is a complex challenge to extrapolate these findings directly to developing countries due to the different nature of their healthcare systems, income distribution and demographic structure [10].

A frequent issue regarding the analysis of cost and inactivity effects of mental disorders is its multicausality nature, as individuals with mental disorders are more susceptible to other diseases such as hypertension and diabetes [11]. Despite the difficulty in establishing a causal mechanism between mental and physical health problems, the presence of a physical disorder has been shown to increase the deleterious effect of mental disorders, especially regarding morbidity and mortality, modifiable lifestyle factors, and access to and quality of healthcare treatment [12].

The Metropolitan Region of São Paulo, Brazil, is a propitious region to analyze the distribution of mental disorders and its effects. From the sample of 28 countries that compose the World Mental Health (WMH) Survey Initiative, a multicentric study coordinated by the WHO, São Paulo was found to have one of the highest prevalence of mental disorders (a total of 10 % of its residents suffered from a recently active severe mental disorder) [13]. São Paulo also has a wide distribution of income and a large racial diversity [14], which mimics the path that most countries are heading to at the beginning of the 21st century.

The objective of the present study is to examine the association of mental disorders with overall health expenditure and lost days of normal activity, by analyzing a representative sample of the Metropolitan Region of São Paulo, Brazil. We also include two secondary objectives: to test if the two most prevalent chronic diseases (hypertension and diabetes) affect the previous relationship, and to describe the most important sources of incremental direct health expenditures.

## Methods

We analyzed data from the São Paulo Megacity Mental Health Survey, a representative sample survey of individuals aged 18 years and older living in the Metropolitan Region of São Paulo. It is part of the World Mental Health (WMH) Survey Initiative, a multicentric study coordinated by the World Health Organization and performed in 28 countries. Data collection was carried out between May 2005 and April 2007. Individuals were selected from a stratified multistage area probability sample of residents of private households living in the metropolitan region, following the same methodology as previous WMH studies [15]. Six stages of selection were employed in order to target 5,000 households from the two geographic strata of the Metropolitan Region of São Paulo (the municipality of São Paulo and its neighboring 38 municipalities). For each household, one resident was randomly selected by the use of a Kish table. The questionnaire was then administered in face-to-face interviews conducted by professional interviewers under the guidance and close supervision of the research team. More details about the sampling methodology are available elsewhere [15].

For the present study, we included only participants who answered the long-form questionnaire administered to all respondents who met lifetime criteria for any disorder in Part 1, plus a probability subsample of the other respondents. The questionnaire included assessment of risk factors, use of services, use of psychiatric medications, socio-demographic information, and, most importantly for our analysis, health expenditure and lost days of normal activity. A total of 2,920 individuals were included in the present analysis. Area of residence was separated by municipalities (average of 232,751 residents in 2007), or, in the case of residing in the municipality of São Paulo, by its 31 administrative regions (known as *subprefeituras*, average of 355,467 residents), totaling 69 level-2 areas included in the multilevel models.

The instrument used for assessing the presence of mental disorders was the Composite International Diagnostic Interview (WMH-CIDI), a fully structured lay interview that uses the Diagnostic and Statistical Manual of Mental Disorders (DSM-IV) criteria, translated and validated into Portuguese [16]. DSM-IV disorders were identified by taking into account the previous 12 months. Individuals were identified as having any mental disorder based on the DSM-IV assessment of at least one core disorder: major depression, generalized anxiety disorders (GAD), social phobia, mania, substance use disorders, panic disorder, specific phobia, agoraphobia, adult separation anxiety (ASA), intermittent explosive disorder (IED), bipolar disorder I and II, attention-deficit/hyperactivity disorder, dysthymia, oppositional-defiant disorder, obsessive-compulsive disorder (OCD), post-traumatic stress disorder (PTSD) and conduct disorder, as well as suicidal behavior. Anxiety disorders were based on the DSM-IV diagnosis of at least one of the following: panic disorder, specific phobia, GAD, agoraphobia without panic disorder, social phobia, PTSD, OCD and ASA. Depression was based on the presence of a major depression episode according to DSM-IV criteria.

Multilevel models were used in order to account for the non-independence of observations within groups, as a previous analysis from the same sample showed that there is a difference in mental disorders prevalence by area of residence [14, 17]. The analysis of expenditure and lost days of work as dependent variables is an issue for the standard ordinary least squares analysis because of its highly skewed distribution, especially given the large number of values equal to zero [9]. We address this problem by using generalized least squares (GLS), a popular methodology for analyzing expenditure data as the dependent variable [8]. All the multilevel models were estimated with MLwiN V.2.25 software, and the descriptive analysis was performed with Stata 13.

We used socioeconomic characteristics of the individuals as control variables. Age was divided into four categories (18–34, 35–49, 50–64 and 65 or more). Education was defined according to the Brazilian system into basic (8 years or less), high school (9–11 years) and college (12 years or more). Marital status was defined as married, single (never married) and previously married (either widowed or divorced). Hypertension and diabetes (the two most frequent chronic diseases in the study) were included to control for its confounding effect, as previous studies have shown its association with both mental disorders and health expenditures [18], and to compare its effects on health costs with the effect of mental disorders. Hypertension and diabetes were assessed with a standard chronic disorders checklist. Lost days of normal activity was assessed by asking the individual: “During the last 12 months, how many days approximately were you *completely* incapable of working or performing your normal daily activities due to health problems?”. Individual income, per capita income of area of residence (a Level 2, or contextual, variable) and health expenditure are presented in terms of Brazilian Reais (R$) in the tables, but also converted throughout the manuscript into US Dollars (US$) calculated first by direct conversion (using the conversion rate of the end of the data collection, i.e. April of 2007), and then by adjusting for the purchasing power parity conversion (PPP) factor from the World Bank [19].

Direct health expenditure excludes payments of healthcare plans and was assessed by the sum of total payments for medications, hospitalizations, medical and other health professional visits, nursing home care, health exams, orthopedic and other medical supplies and other health services and supplies. The questionnaire for health expenditure referred to the last three months, but the results were standardized for annual values to allow comparability with other studies and with the annual values used for lost days of normal activity. We analyzed the results according to the presence of any mental disorder (at least one recently active disorder), and also separately for depression and anxiety, which were the two most prevalent categories of mental disorders identified by the study. Results were presented according to the guidelines of the STrengthening the Reporting of OBservational studies in Epidemiology (STROBE) statement [20]. The data set supporting the results of this article is available in the LabArchives repository, https://dx.doi.org/10.6070/H4XK8CH0 [21].

We first present the descriptive table, with the distribution of the variables for the total sample and for each of the mental disorders. We then show the results of the multilevel models separately for health expenditure and lost days of normal activity. Both tables follow the same procedure: a null model (Model 0) to compare model fit (using the log likelihood), Model 1 includes the sociodemographic control variables, Model 2 is the same but for the inclusion of depression, Model 3 includes anxiety and Model 4 any mental disorders. We follow these two tables by an analysis of the potential effect of the two most frequent chronic diseases (hypertension and diabetes) in changing the previous results. We then also analyze the separate effect of costs from medical visits and medication.

### Ethics statements

The SPMHS procedures for recruitment, obtaining informed consent, and protecting human subjects during field procedures were approved by the Research and Ethics Committee of the University of São Paulo Medical School. Respondents were interviewed only after informed written consent was obtained, and total confidentiality was assured. Eligible respondents were those who were 18 or older, Portuguese-speaking, and without any disability or handicap that would otherwise impair their ability to participate in the interview.

## Results

Table 1 presents the distribution of the variables for the total sample and according to mental disorders. Females were 57.9 % of the total sample, but 75.2 % of the individuals with depression, 69.6 % of the ones with anxiety and 66.0 % of the ones with any mental disorder. The distribution of age, education and marital status was similar for the total sample in relation to the ones with mental disorders. Individuals with mental disorders had a slightly higher prevalence of hypertension and diabetes when compared with the total sample, a result that was higher for those with anxiety.

**Table 1:** Distribution of socioeconomic characteristic and the most prevalent chronic diseases according to the presence of mental disorders, São Paulo Megacity Study, Brazil

	Total		Depression		Anxiety		Any mental disorder	
	N	%	N	%	N	%	N	%
Total	2920	100.00	536	100.00	836	100.00	1263	100.00
Sex								
Women	1692	57.95	403	75.19	582	69.62	834	66.03
Age								
18 to 34	1083	37.09	199	37.13	285	34.09	479	37.93
35 to 49	1020	34.93	214	39.93	310	37.08	468	37.05
50 to 64	589	20.17	102	19.03	196	23.44	253	20.03
65 or older	228	7.81	21	3.92	45	5.38	63	4.99
Education								
Basic	1521	52.09	273	50.93	478	57.18	674	53.37
High School	969	33.18	182	33.96	239	28.59	415	32.86
College	430	14.73	81	15.11	119	14.23	174	13.78
Marital status								
Single	948	32.47	179	33.40	246	29.43	423	33.49
Widowed/divorced	613	20.99	126	23.51	212	25.36	287	22.72
Married	1358	46.51	231	43.10	378	45.22	553	43.78
Missing	1	0.03	0	0.00	0	0.00	0	0.00
Individual income								
Median	4687.20	–	4684.00	–	4686.00	–	4684.00	–
Private health plan								
Yes	1119	38.32	203	37.87	309	36.96	466	36.90
Per capita income (contextual)								
Median	683.50	-	727.02	-	696.62	-	718.09	-
Hypertension								
Yes	706	24.18	141	26.31	247	29.55	331	26.21
Missing	13	0.45	4	0.75	6	0.72	8	0.63
Diabetes								
Yes	200	6.85	43	8.02	69	8.25	100	7.92
Missing	30	1.03	10	1.87	16	1.91	20	1.58

Table 2 shows the determinants of direct health expenditure. For the model without mental disorders (Model 1), being female was statistically associated with an incremental annual health expenditure of R$110.68 (US$90.64 in terms of PPP) with a 95 % Confidence Interval (95 % CI) of R$20.4-R$200.95. Being older was also statistically associated with higher expenditure in relation to the 18–34 years old group. Having a healthcare plan was also independently associated with higher direct expenditure. The contextual variable (per capita income of area of residence) was also associated with a small, but statistically significant, health expenditure. The model with better overall fit (lower log likelihood) was Model 2. Depression (Model 2) was associated with an incremental annual expenditure of R$308.28 (US$252.48), 95%CI: R$194.05-R$422.50. Anxiety and any mental disorder (Models 3 and 4) were also associated with a statistically significant higher expenditure, of R$177.82 and R$180.52, respectively (or US$145.64 and US$147.85).

**Table 2:** Multilevel models of the determinants of annual health expenditure, São Paulo Megacity Study, Brazil

	Null model	Model 1		Model 2		Model 3		Model 4	
		β	95 % CI	β	95 % CI	β	95 % CI	β	95 % CI
Sex (women)		110.68	20.41–200.95	71.31	−19.71–162.33	87.68	−3.28–178.65	86.11	−4.74–176.96
Age									
18 to 34									
35 to 49		116.68	5.05–228.31	107.75	−3.39–218.89	113.38	1.98–224.78	114.42	3.10–225.75
50 to 64		225.95	86.71–365.19	227.12	88.55–365.70	219.80	80.82–358.78	229.11	90.23–367.99
65 or older		398.88	207.47–590.28	426.35	235.58–617.12	418.46	227.16–609.77	432.40	240.78–624.02
Education									
Basic									
High School		85.59	−21.26–192.44	84.49	−21.85–190.83	96.18	−10.60–202.96	91.05	−15.55–197.65
College		121.98	−30.23–274.18	120.66	−30.82–272.14	127.36	−24.54–279.27	130.52	−21.34–282.38
Marital status									
Single									
Widowed/divorced		68.34	−68.02–204.71	67.00	−68.73–202.72	58.18	−78.02–194.38	64.73	−71.29–200.74
Married		45.54	−64.23–155.30	49.20	−60.05–158.45	43.55	−65.98–153.08	50.23	−59.27–159.73
Individual income		0.01	0.00–0.01	0.01	0.00–0.01	0.01	0.00–0.01	0.01	0.00–0.01
Private health plan		283.96	185.49–382.43	285.02	187.02–383.02	283.99	185.73–382.25	285.28	187.06–383.49
Per capita income (contextual)		0.21	0.13–0.29	0.21	0.13–0.29	0.21	0.13–0.29	0.21	0.13–0.21
Depression				308.28	194.05–422.50				
Anxiety						177.82	79.68–275.97		
Any mental disorder								180.52	91.13–269.92
Log likelihood	49887.88	49695.48		49667.64		49682.89		49679.86	

Table 3 presents the determinants of lost days of normal activity. For the model with only the socioeconomic variables, being older was associated with more missing days of normal activity, but education, marital status and having a healthcare plan were not. Depression (Model 2) was statistically associated with an incremental 10.37 lost days of normal activity (95 % CI: 6.31–14.42). For this model, being a woman was associated with a decrease of 4.00 days of lost normal activity (95 % CI: −7.22 – −0.77). Anxiety and any mental disorders (Models 3 and 4) were also statistically associated with more lost days of normal activity of 4.20 and 4.25 respectively (95 % CI: 0.72–7.69 and 1.08–7.43).

**Table 3:** Multilevel models of the determinants of lost days of normal activity, São Paulo Megacity Study, Brazil

	Null model	Model 1		Model 2		Model 3		Model 4	
		β	95 % CI	β	95 % CI	β	95 % CI	β	95 % CI
Sex (women)		−2.67	−5.87–0.53	−4.00	−7.22–−0.77	−3.22	−6.45–0.02	−3.25	−6.48–−0.02
Age									
18 to 34									
35 to 49		5.02	1.06–8.98	4.72	0.78–8.66	4.94	0.99–8.90	4.97	1.01–8.92
50 to 64		7.19	2.25–12.12	7.22	2.31–12.14	7.04	2.11–11.97	7.27	2.34–12.20
65 or older		6.30	−0.48–13.09	7.23	0.46–14.00	6.77	−0.03–13.56	7.09	0.29–13.90
Education									
Basic									
High School		−2.51	−6.30–1.28	−2.55	−6.32–1.22	−2.26	−6.05–1.53	−2.39	−6.17–1.40
College		−4.51	−9.91–0.89	−4.56	−9.93–0.82	−4.39	−9.78–1.01	−4.31	−9.70–1.08
Marital status									
Single									
Widowed/divorced		−1.01	−5.84–3.83	−1.05	−5.87–3.77	−1.25	−6.08–3.59	−1.09	−5.92–3.74
Married		−0.39	−4.28–3.51	−0.26	−4.14–3.61	−0.43	−4.32–3.46	−0.28	−4.16–3.61
Individual income		0.00	0.00–0.00	0.00	0.00–0.00	0.00	0.00–0.00	0.00	0.00–0.00
Private health plan		3.04	−0.45–6.53	3.08	−0.40–6.56	3.04	−0.45–6.53	3.07	−0.41–6.56
Per capita income (contextual)		−0.01	−0.02–0.00	−0.01	−0.02–0.00	0.00	−0.01–0.00	−0.01	−0.02–0.00
Depression				10.37	6.31–14.42				
Anxiety						4.20	0.72–7.69		
Any mental disorder								4.25	1.08–7.43
Log likelihood	30237.60	30200.21		30175.17		30194.62		30193.32	

Table 4 includes hypertension and diabetes on the last models (Model 4) of the two previous tables. Even with the inclusion of hypertension and diabetes (and a third new model with both chronic diseases), depression, anxiety and any mental disorders were still statistically associated with an incremental higher health expenditure and missing days of normal activity, despite the fact that the coefficients were somewhat smaller in relation to the previous models. More interestingly, depression was independently associated with a higher health expenditure than hypertension and diabetes. For Model 3 (that includes diabetes and hypertension), depression was associated with an incremental expenditure of R$286.33 (US$234.50), while for hypertension and diabetes it was R$188.00 and R$127.28, respectively (US$153.97 and US$104.24; results not shown).

**Table 4:** Multilevel models with the inclusion of the two most prevalent chronic diseases (hypertension and diabetes), São Paulo Megacity Study, Brazil

	Model 1 (with hypertension)		Model 2 (with diabetes)		Model 3 (hypertension and diabetes)	
	β	95 % CI	β	95 % CI	β	95 % CI
Health expenditure						
Depression	290.18	176.13 – 404.24	290.72	175.65 – 405.79	286.33	170.96 – 401.69
Anxiety	159.82	61.74 – 257.89	163.99	65.27 – 262.70	155.22	56.14 – 254.30
Any mental disorder	163.79	74.55 – 253.03	164.30	74.43 – 254.17	158.74	68.65 – 248.83
Lost days of normal activity						
Depression	9.58	5.55 – 13.61	9.74	5.73 – 13.76	9.70	5.67 – 13.73
Anxiety	3.57	0.10 – 7.03	3.94	0.49 – 7.38	3.78	0.32 – 7.25
Any mental disorder	3.78	0.62 – 6.93	4.05	0.91 – 7.19	3.94	0.79 – 7.09

The fifth and last table (Table 5) shows health expenditures from medication and medical visits separately (the two highest sources of health expenditure from the sample), also by building upon the last models of Tables 2 and 3. When analyzing the final models with hypertension and diabetes, depression was associated with an incremental expenditure of R$193.49 (US$158.47) from medication and R$73.22 (US$59.97) from medical visits. The association of anxiety and expenditure with medication was statistically significant, but for medical visits it was not. For any mental disorders, both medication and medical visits were significant, leading to a higher health expenditure of R$111.82 (US$91.58) and R$39.76 (US$32.56), respectively.

**Table 5:** Multilevel models of health expenditure separately for medication and medical visits, São Paulo Megacity Study, Brazil

	Medications		Medical visits	
	β	95 % CI	β	95 % CI
Depression				
Without chronic diseases	214.36	149.06 – 279.65	71.86	45.34 – 98.37
With hypertension and diabetes	193.49	128.35 – 258.63	73.22	46.20 – 100.25
Anxiety				
Without chronic diseases	140.58	84.47 – 196.69	20.51	−2.31 – 43.33
With hypertension and diabetes	118.62	62.65 – 174.59	20.44	−2.81 – 43.70
Any mental disorder				
Without chronic diseases	128.38	77.24 – 179.52	39.15	18.39 – 59.91
With hypertension and diabetes	111.82	60.92 – 162.72	39.76	18.65 – 60.88

## Discussion

Depression, anxiety and any mental disorders were consistently associated with both higher expenditure for overall health problems and lost days of normal activity. This association remained robust even when the most prevalent chronic diseases (hypertension and depression) were included in the multilevel models. More interestingly, depression was associated with a higher incremental health expenditure than hypertension and diabetes, two common chronic diseases that are easily diagnosed and amenable to treatment. The higher health expenditure found for individuals with mental disorders is particularly notable given the fact that Brazil has a universal free-of-charge coverage healthcare system. While individual access to public hospitals and free medication is an issue for most parts of Brazil, the Metropolitan Region of São Paulo has a wide distribution of public hospitals and medication distribution centers [22].

When hypertension and diabetes were included in the models, depression, anxiety and any mental disorders were still associated with a statistically significant health expenditure of US$234.50, US$127.13 and US$130.01, respectively. The majority of the expenditure came from medication (US$158.47, US$97.15 and US$91.58, respectively), but expenditure from medical visits was also statistically significant for depression and for any mental disorders (US$59.97 and US$32.57, respectively). The importance of medication in relation to total expenditure could be related to structural problems of healthcare system in Brazil. The country is well known for its long waiting queue in order to get an appointment with a physician, even in areas well-served by medical services. This fact associated with the possibility of obtaining over-the-counter medications for the majority of chronic conditions could explain our findings.

Despite previous studies that showed that the presence of mental disorders was strongly correlated with higher use of health care services in São Paulo [22], there is a possibility that the prevailing stigmas against mental disorders and the low accessibility to specialty care could push a large number of individuals to prefer self-medication instead of formal clinical visits [23]. Although both anxiety and any mental disorders were significantly associated with higher health expenditure and lost days of normal activity, our analyses showed that depression was associated with higher direct health costs. Its independent effect on health expenditure surpassed hypertension and diabetes by US$80.55 and US$130.26, respectively.

Lost days of normal activity were also statistically associated with the presence of depression, anxiety and any mental disorders. As with health expenditure, depression was associated with a higher incremental effect than anxiety, for which an association had already been described in a previous analysis [24]. On the final multilevel model, depression was associated with an annual 9.70 days of being completely unable to perform normal activities or working. That number was smaller for anxiety and any mental disorders (3.78 and 3.94), but it was still statistically significant. This adds an extra burden to the direct expenditure, as missing days of work are associated with loss of productivity and lower wages [7].

Brazil has a poor history of dealing with mental disorders, and has considerably fewer psychiatrists per residents than developed countries [4]. One important strength of our study was that it did not simply ask individuals if they had ever been diagnosed with a mental disorder, but used the WMH-CIDI, a fully structured lay interview, to identify its presence. There is a growing debate on the literature if the recent increase in the burden of mental health is solely due to an increase in diagnosis or to an actual higher number of individuals with mental disorders [25]. Given the fact that Brazil still has a poor access to mental healthcare, the use of self-referred data on mental disorders could have significantly underestimate its presence. On the other hand, even a validated questionnaire such as WHM-CIDI has its limitation due to local cultural differences in admitting some of the emotional symptoms and substance use problems [26].

We note other limitations of our study. First, despite having a relatively acceptable response rate (81.3 %), the presence of response bias cannot be discarded. Second, it was based on a cross-sectional survey, so the temporal association between mental disorders and health expenditure and lost days of normal activity was not established. Third, the sampling strategy was based on area probability, which means that each of the separate individual characteristics were not individually representative of the population. Fourth, it could be argued that another limitation is that individuals were asked about health expenditure and lost days of normal activity in terms of any disease and not specifically for mental disorders, but even if this question was available, we would still argue that an overall analysis is more appropriate due to the multicausality of mental disorders and the challenge that is identifying the importance of mental disorders as the determining cause of a lost day of normal activity or a healthcare expenditure.

## Conclusion

Our analysis found a robust association of depression, anxiety and any mental disorder with incremental health expenditure and lost days of normal activity, even in a country with a universal free-of-charge healthcare system. These results highlight the growing importance of mental disorders as a defining public health issue for developing countries, not only in terms of individual wellbeing but also in terms of its economic and societal consequences. Expanding public mental healthcare coverage could help to decrease individual health expenditures in Brazil, but will require a public health commitment from the government and a change from the current stigma against mental disorders.
